# Effects of a leadership-focused implementation strategy on uptake of digital measurement-based care in mental health clinics: a cluster randomized trial

**DOI:** 10.1093/tbm/ibag007

**Published:** 2026-03-13

**Authors:** Nathaniel J Williams, Gregory A Aarons, Mark G Ehrhart, Nallely Vega, Steven C Marcus

**Affiliations:** School of Social Work, Boise State University, Boise, ID, United States; Department of Psychiatry, University of California, San Diego, CA, United States; Department of Psychology, University of Central Florida, Orlando, FL, United States; School of Social Work, Boise State University, Boise, ID, United States; The Ballmer Institute for Children’s Behavioral Health, University of Oregon, Portland, OR, United States; School of Social Policy and Practice, University of Pennsylvania, Philadelphia, PA, United States

**Keywords:** organizational leadership, evidence-based practice, uptake, implementation, sustainment, measurement-based care, leadership and organizational change for implementation, WISDOM trial

## Abstract

**Background:**

Leaders of healthcare organizations are often called on to guide the implementation of new innovations, including evidence-based practices and digital health technologies. However, many leaders lack preparation for this role and most available leadership trainings have not been rigorously tested, particularly over periods incorporating multiple implementation phases.

**Purpose:**

This study tested the effects of the Leadership and Organizational Change for Implementation (LOCI) strategy on the uptake of digital measurement-based care (MBC) in mental health settings across 35 months, incorporating implementation and sustainment phases.

**Methods:**

In 21 outpatient mental health clinics serving youth, a two-arm, cluster randomized, hybrid type III effectiveness-implementation trial tested whether adding LOCI (*k* = 11) to standard digital MBC training and technical assistance (*k* = 10) improved uptake of digital MBC, assessed using system generated data and operationalized as clinic-level monthly counts of the number of youths with a measure administered and with feedback viewed by their clinician.

**Results:**

On both outcomes, clinics randomized to LOCI exhibited superior initial uptake (3 months post-baseline: mean difference in youths with measure administered per clinic (*M_YMA_Diff_*=5.09, 95% CI=[1.63–8.55]); mean difference in youths with feedback viewed per clinic (*M_YFV_Diff_*=3.81 [1.26–6.37]), superior uptake when the implementation phase concluded (13 months post-baseline: *M_YMA_Diff_*=9.03, [1.64–16.41]; *M_YFV_Diff_*=8.31 [3.07–13.56]), and superior uptake when the sustainment phase concluded (35 months post-baseline: *M_YMA_Diff_*=3.82 [1.28–6.36]; *M_YFV_Diff_*=1.41 [0.22–2.60]).

**Conclusions:**

LOCI is an effective approach for training organizational leaders to support implementation of evidence-based digital health technologies in healthcare settings. Studies examining how policy-level variables interact with leadership training are needed.

## Teaser

Adding a leadership-focused strategy to standard training and technical assistance significantly improved the implementation and sustainment of a new health technology over nearly three years.

Implications **Practice:** Leaders of healthcare organizations can improve their organization’s effectiveness in implementing new evidence-based digital health technologies by participating in empirically-supported, leadership training and consultation strategies like Leadership and Organizational Change for Implementation (LOCI).

**Policy:** Funders and policymakers should prioritize organizational leadership training in campaigns to implement new evidence-based digital health technologies.

**Research:** Researchers should ensure that implementation studies take into account, through measurement, implementation strategies, and/or design, potentially important variation across sites in implementation leadership.

## Introduction

Numerous studies document the low rates at which evidence-based practices (EBPs) and evidence-based digital health technologies are adopted and sustained over time in mental health settings when traditional implementation strategies, such as clinician training, are the sole source of support [1]. Barriers to implementation occur at multiple levels, and while some are EBP-specific, one of the most frequently cited and influential challenges across EBPs and sites involves gaps in the preparation of organizational leaders [2, 3], including among first-level leaders, who serve as clinical and managerial supervisors of clinicians [4–7], and executive leaders, who establish the organization’s mission, strategy, and operations [7, 8]. Many leaders in mental health settings are clinicians promoted into leadership roles because of their clinical competence and professional longevity [9]. These leaders may have excellent clinical acumen and other skills but are rarely trained or equipped to effectively lead organizational and practice change, which is essential when implementing new EBPs and digital health technologies [10–12]. Given the potentially significant impact leaders have on implementation success [8], training and equipping them represents a potentially powerful leverage point; however, very few implementation trials test strategies that target leaders and consequently little is known about the best ways to support them [13–17].

Digital measurement-based care, which involves administering web-based, patient-reported outcome measures prior to each clinical encounter and using the feedback to guide treatment [18], is an EBP that has faced considerable barriers to widespread implementation and sustainment [19, 20] despite robust evidence of effectiveness and high generalizability across mental health patient populations and settings [21–24]. Studies cataloging barriers and facilitators to implementing digital measurement-based care consistently highlight the importance of organizational factors, particularly organizational leadership, as a key determinant of failure or success [19, 25–29]. Despite this, the few trials addressing measurement-based care implementation to date have focused on training clinicians rather than organizational leaders [19, 30] and no trials prior to the present one have examined issues of sustainment.

In this study, we examined the effects of a leadership-focused organizational implementation strategy, called Leadership and Organizational Change for Implementation (LOCI) [31–34], on the uptake of digital measurement-based care in community mental health clinics across 35 months. The LOCI strategy includes quarterly trainings and brief weekly coaching for first-level leaders, quarterly organizational strategy meetings attended by organizational executives and first-level leaders, and monthly planning meetings for executives, supplemented by data-based feedback on leadership and climate collected from clinicians. The goal of these activities is to align leadership across levels, equip leaders with focused implementation leadership skills, and provide a framework for generating a focused organizational implementation climate that conveys strong expectations, support, and rewards for the use of a specific EBP. Improvements in organizational implementation leadership and climate are in turn expected to speed and increase the uptake of the EBP during the implementation phase [35], while also contributing to superior uptake during the sustainment phase as organizational policies, procedures, and practices become embedded.

Drawing on the Exploration, Preparation, Implementation and Sustainment (EPIS) model [36, 37], and on empirical research examining implementation phases [38–41], we divided the study’s nearly 3-year timeframe into three phases: implementation (immediately after measurement-based care training and while the LOCI strategy was active), post-implementation (following the conclusion of LOCI but prior to recommended timeframes for examining sustainment), and sustainment (from 24 months after measurement-based care training to the study endpoint). This study examines the population-level impact of LOCI on the uptake of digital measurement-based care over time using previously unreported clinic-level data from the Working to Implement and Sustain Digital Outcome Measures (WISDOM) trial [32, 33]. Prior reports on the WISDOM trial described clinical outcomes and youth-level implementation outcomes for a subset of youth patients [32, 33]. In this study, we examine LOCI’s short- and long-term effects on clinician and staff uptake of digital measurement-based care, which is an essential bridge to ultimately achieving fidelity and improved clinical outcomes for youths [42, 43].

In view of LOCI’s theory of change and the EPIS model, we tested whether adding LOCI to standard digital measurement-based care training and technical assistance would improve uptake, operationalized as the number of youth with measures administered and feedback reports viewed by their clinicians, in community mental health clinics. Focusing on the implementation and sustainment phases described above, we hypothesized that, compared to clinics receiving training and technical assistance only, LOCI clinics would exhibit greater uptake at the beginning and end of each phase, would demonstrate faster growth in uptake during the implementation phase, and would demonstrate slower deterioration in uptake during the sustainment phase.

## Methods

### Study design and sample

Details of the WISDOM trial protocol are presented elsewhere [32, 33]. Briefly, the trial employed a two-arm, cluster randomized, hybrid type III effectiveness-implementation design [44] to test training and technical assistance alone (control condition; *k* = 10) versus with the addition of LOCI (LOCI condition; *k* = 11), on the implementation and sustainment of digital measurement-based care. The trial included 21 outpatient mental health clinics delivering psychotherapy to youth, ages 4-17. Within the trial, implementation outcomes were studied at two levels: youth and clinic. Primary youth-level implementation outcomes, including a measurement-based care fidelity index and completion rate, were examined for a subset of participants; these analyses are reported elsewhere [32, 33]. At the clinic level, the focal implementation outcome was uptake as operationalized and reported in this paper.

Clinics were eligible if they were located in Idaho, Oregon, or Nevada, USA; had 3+ full-time equivalent clinicians serving youths; and were not currently implementing a digital measurement-based care system. Both for-profit and not-for-profit community mental health clinics that provided outpatient psychotherapy to youth were included. From June to October 2019, the first 21 clinics that agreed to participate were enrolled. Using covariate constrained randomization [45] that accounted for clinic size and State, clinics were allocated in a 1:1 ratio by the study statistician to LOCI or control conditions. The first LOCI training for clinic leaders occurred in November 2019. Training in digital measurement-based care for clinicians and leaders in both conditions occurred one month later. One month after measurement-based care training, the system began collecting data on all measures administered and feedback viewed, which were aggregated to the clinic-level for analysis. Clinicians, youths, and caregivers, but not clinic executives or first-level leaders, were masked to study condition. Ethics approval and oversight were provided by the affiliated Institutional review board. The CONSORT [46] and StaRI [47] checklists were followed for reporting of this trial.

### Digital Measurement-Based care

The digital measurement-based care system used in this trial was the Outcomes Questionnaire Analyst (OQ-A) [48, 49]. All clinics received access to the web-based OQ-A system for the duration of the study at no cost. The system included a brief measure of youth symptoms and functioning, the Youth Outcomes Questionnaire 30.2 [50, 51], to be completed by youth (ages 12–18) and/or their caregivers prior to every treatment session, and a Treatment Support Measure [52, 53], completed at the clinician’s discretion by either youth and/or their caregivers, to assess potential barriers to clinical progress (e.g. therapeutic alliance, social support). Measures were available in English and Spanish and took approximately 4 minutes to complete. Clinics developed tailored protocols guiding who administered measures (e.g., front desk staff, clinicians) and in what format (e.g. tablet in the waiting room versus secure link sent to participants’ phones). The study protocol called for clinicians to view feedback prior to or within 7 days of each session, to share feedback with youth and families as they deemed clinically appropriate, and to use the feedback to inform clinical decision-making.

### Implementation strategies

All clinicians and organizational leaders working with youth in both conditions received an initial one-day in-person training in digital measurement-based care provided by the OQ-A purveyor organization. This was followed by two, 1-hr, live, web-based booster trainings approximately 3 and 5 months later. After initial training, all clinics received technical assistance provided by the OQ-A organization, including: on-demand web-based trainings; a library of training videos; manuals; and, technical support for the duration of the study.

Leaders in LOCI clinics participated in the LOCI leadership training strategy for 12 months. First-level leaders completed quarterly trainings (30 total hours), weekly coaching calls (∼20 minutes each), and participated in quarterly 1-hr organizational strategy meetings with organizational executives and the LOCI team to enhance organization and clinic alignment for implementation planning. Approximately once per month, first-level leaders’ weekly coaching calls were replaced by a 1-hour group coaching call with all LOCI leaders, to facilitate sharing of ideas across clinics. Executive leaders participated in quarterly organizational strategy meetings (1-hour) and, in-between the strategy meetings, monthly planning meetings (30–60 minutes). Quarterly leadership trainings occurred in a group setting incorporating leaders from all LOCI clinics. For the first two quarters, LOCI leadership trainings and organizational strategy meetings occurred in-person; however, due to restrictions associated with the COVID-19 pandemic, events were held virtually thereafter. Monthly meetings with executives occurred virtually. Supplemental Table 1 describes the number of LOCI activities completed by leaders overall and by clinic.

Guided by individualized leadership development plans and clinic-wide climate development plans, and informed by survey data on leadership and climate collected from clinicians, clinic leaders applied strategies to shape the entire process of digital measurement-based care implementation from initial administration of measures to review of feedback by clinicians. During the final quarter of LOCI, activities began to address sustainment by encouraging leaders to add goals to their leadership development plans and climate development plans addressing the supports needed to ensure continued use of digital measurement-based care after LOCI concluded. Williams *et al.* [32] provide a detailed description of the LOCI strategy applied in this trial.

### Study outcomes

We examined uptake of digital measurement-based care using two monthly, clinic-level, count outcomes, each capturing distinct aspects of the measurement-based care process. The first was the count of the number of youth in each clinic who had a Youth Outcomes Questionnaire measure administered. The second was the count of the number of youth whose clinician viewed the Youth Outcomes Questionnaire feedback report. Feedback review is the second step of the measurement-based care process and a common point at which implementation breaks down, even in well-funded trials [29, 54, 55]. Both outcomes addressed the Youth Outcomes Questionnaire because the study protocol called for its session-by-session administration and review, whereas administration of the Treatment Support Measure was discretionary, dependent upon clinical circumstances, and exhibited minimal variability. Data on both outcomes were derived from the OQ-A system which automatically logged every occurrence of measure administration and feedback viewing. A youth was counted as having a measure administered in a given month if either the youth or caregiver completed a Youth Outcomes Questionnaire. A youth was counted as having a feedback report viewed if the system logged a clinician accessing one or more of the youth’s feedback reports during the month. Both outcomes were generated for each clinic monthly for 33 months.

### Data analysis

**Figure 1:**
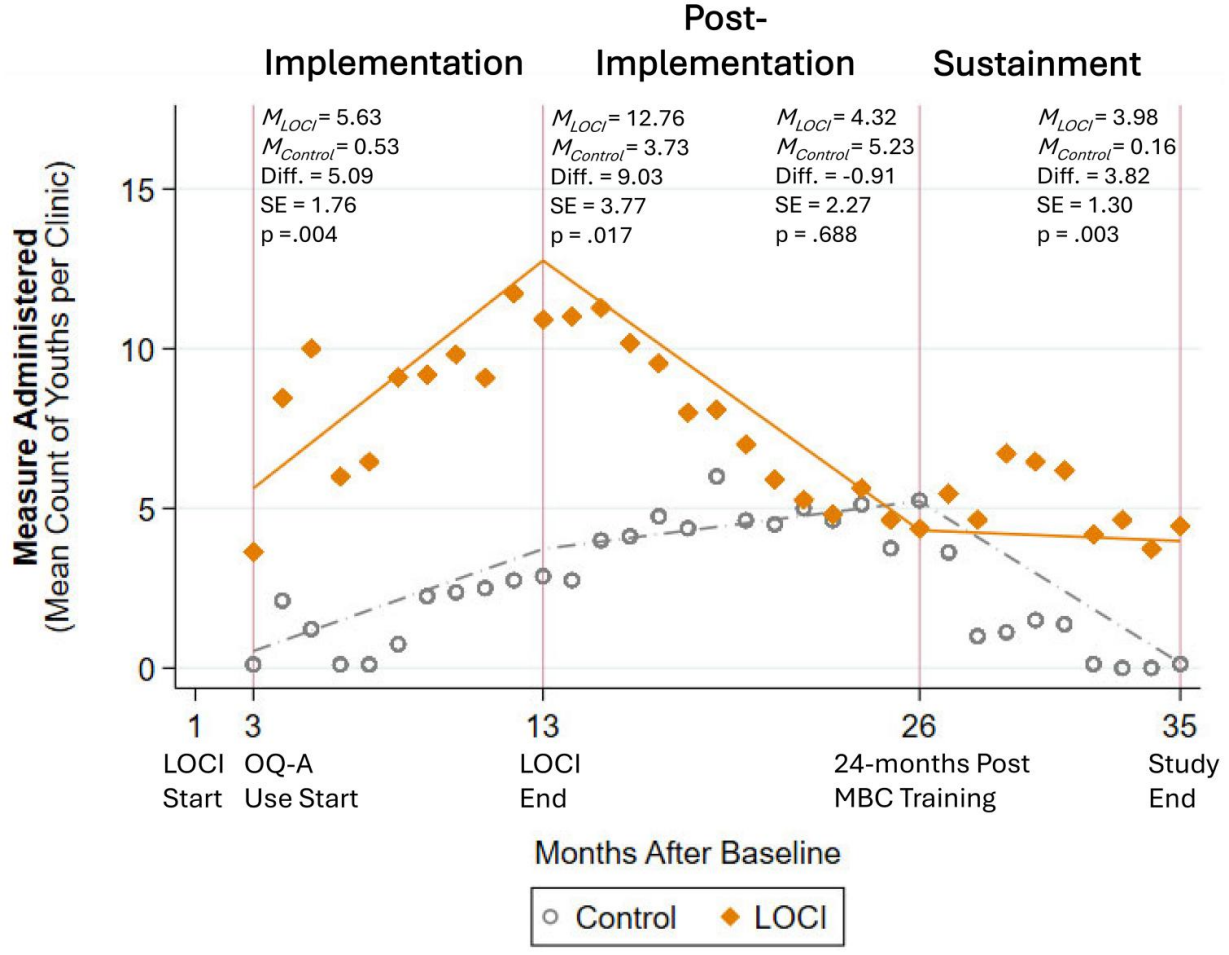
Effects of the leadership and organizational change for implementation (LOCI) strategy on measurement-based care uptake (measure administration) across implementation phases. *Note: N*=632 observations (*K*=20 clinics). Diamonds and dots represent raw means (of counts) by condition by month. Lines indicate model estimated predictive margins (adjusted counts) by condition by phase. MBC = measurement-based care; M_C_ = model-estimated mean count for control condition; M_L_ = model-estimated mean count for LOCI condition; OQ-A = Outcomes Questionnaire-Analyst.

**Figure 2:**
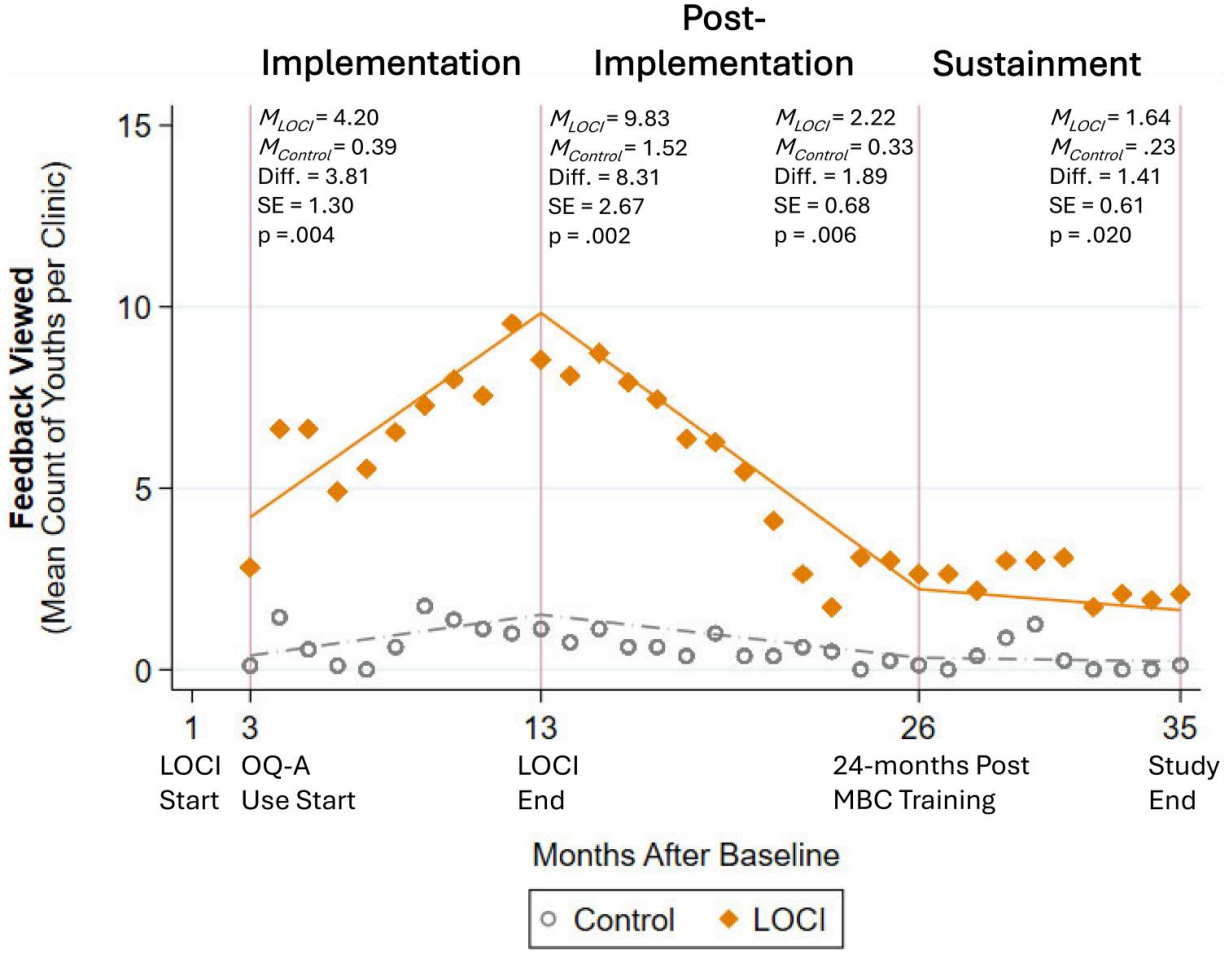
Effects of the leadership and organizational change for implementation (LOCI) strategy on measurement-based care uptake (feedback viewing) across implementation phases. *Note: N*=632 observations (*K*=20 clinics). Diamonds and dots represent raw means (of counts) by condition by month. Lines indicate model estimated predictive margins (adjusted counts) by condition by phase. MBC = measurement-based care; M_C_ = model-estimated mean count for control condition; M_L_ = model-estimated mean count for LOCI condition; OQ-A = Outcomes Questionnaire-Analyst.

Given the longitudinal data structure, incorporation of count outcomes, and our interest in change over time, intent-to-treat analyses were conducted using generalized estimating equations with a negative binomial distribution, log link, and an autoregressive working correlation structure (AR 1) to model marginal changes in outcomes by condition over time [56]. Time was specified using a piecewise function [57] corresponding to the three implementation phases shown in Figures 1 and 2: implementation (3–13 months after baseline, concurrent with LOCI), post-implementation (13-26 months after baseline, from the end of LOCI to 24 months post-training in measurement-based care), and sustainment (26–35 months after baseline, from 24 months post-training to the study endpoint). To account for variation in clinic size, all models adjusted for the number of youth served in the year prior to the trial. One clinic permanently closed five months after the trial began for reasons unrelated to the study, resulting in 28 missing clinic-month observations. Under these conditions, generalized estimating equations provide consistent marginal regression estimates when missingness is missing at random conditional on observed covariates included in the model (i.e., covariate-dependent MAR) [58], an assumption that is plausible given the exogenous nature of the clinic closure and the inclusion of the model covariates. To assess the potential of extreme outliers to unduly influence the results, we calculated Pearson residuals across all 33 observations for each clinic, generated the mean absolute residual per clinic, and identified clinics with mean Pearson residuals > 2, which represents extreme misfit and undue influence. Only one clinic had an absolute mean Pearson residual >2 and its value of 2.4 was 3.5 times greater than the average residual for all other clinics. This extreme clinic outlier was excluded from analysis. Analyses were implemented using xtgee in Stata version 17 [59]. We used the margins command to compare model-estimated counts (predictive margins) by condition at the beginning and end of each phase, and tested time-by-condition interaction terms from the models to assess differences in slopes. The criterion for statistical significance was set at alpha = 0.05.

## Results

Table 1 presents characteristics of the sample clinics at baseline. Supplemental Figure 1, available online, presents a CONSORT diagram showing the flow of clinics through the trial for this analysis.

**Table 1:** Clinic sample characteristics (*K* = 20).

Characteristic	MBC TTA only (*K* = 9)		LOCI + MBC TTA (*K* = 11)	
	*M*	*SD*	*M*	*SD*
Number of youth served in prior year	330.2	174.9	433.1	298.1
Number of full-time equivalent clinicians	8.4	5.0	10.1	6.1
% of youth clients with Medicaid insurance	63.6	29.2	55.6	26.0
Number of EBPs implemented prior to trial	2.7	3.0	2.2	2.7

	*N*	%	*N*	%
State				
Idaho	7	78	7	64
Oregon	2	22	3	27
Nevada	0	0	1	9
Legal status				
Non-profit	3	33	5	45
For profit	6	67	6	55
Populations served				
Children only	0	0	1	9
Adults and children	9	100	10	91

EBP = evidence-based practice; LOCI = Leadership and Organizational Change for Implementation strategy; MBC = measurement-based care; TTA = technical training and assistance.

During the implementation phase, we expected LOCI clinics to exhibit faster uptake of digital measurement-based care, with more youths having measures administered and feedback reports viewed on average per clinic at 3 months postbaseline. As is shown in Figs. 1 and 2, this hypothesis was supported for both outcomes. We also anticipated a steeper trajectory of uptake during the implementation phase in LOCI clinics. Although this pattern is visually evident from the raw means graphed in Figs. 1 and 2, the interaction parameter testing the difference in log-linear slopes was not statistically significant for either the average number of youths with a measure administered per clinic (*b *= −0.11, SE = 0.08, *P* = .145) nor the number with feedback viewed per clinic (*b *=*−*0.05, *SE *= 0.08, *P* = .548). Finally, we expected LOCI clinics to exhibit superior uptake at 13 months postbaseline, corresponding to the conclusion of LOCI and the end of the implementation phase. This hypothesis was supported for both outcomes (see Figs. 1 and 2).

During the sustainment phase, we expected LOCI clinics to exhibit superior uptake 24 months after measurement-based care training (26 months postbaseline). This hypothesis was not supported for the count of youths with a measure administered on average per clinic (see Fig. 1), but was supported for the count of youths with a feedback report viewed (see Fig. 2). We also hypothesized a more positive trajectory of change in uptake for LOCI clinics during the sustainment phase. This hypothesis was partially supported. The LOCI clinics had a significantly less steep rate of decline in the count of youths with a measure administered on average per clinic per month (*b *= 0.38, SE = 0.11, *P* = .000; see Fig. 1), but there was no difference between conditions on rate of change in the number of youths with feedback viewed per clinic per month (*b *= 0.01, SE = 0.12, *P* = .947; see Fig. 2). Finally, we expected LOCI clinics to have greater uptake at the study endpoint (35 months post-baseline), and this hypothesis was supported for both outcomes (see Figs. 1 and 2).

## Discussion

These secondary analyses of the WISDOM trial fill knowledge gaps regarding how best to support organizational leaders in facilitating implementation of evidence-based digital health technologies by testing the LOCI strategy’s effects on population-level uptake of digital measurement-based care across implementation and sustainment phases. Consistent with our hypotheses, clinicians and staff in LOCI clinics exhibited faster initial uptake and greater total uptake at the height of the implementation phase, even though there was not a statistically significant difference in the slopes linking these points. The lack of a statistically significant difference in the log-linear slopes during the implementation phase suggests the trajectories of change were similar for the two conditions during this period even though the LOCI clinics exhibited 10.6 times greater uptake in measure administration and 10.8 times greater uptake in feedback viewing by 3 months postbaseline, followed by 3.4 times greater and 6.5 times greater uptake on these outcomes, respectively, at 13 months postbaseline. The pattern of quicker onset and greater uptake in LOCI clinics, evident for both outcomes, suggests the LOCI strategy had broad and positive impacts on digital measurement-based care delivery during the implementation phase.

The most important finding from the sustainment phase was that the number of youth with measures administered and feedback viewed was significantly higher in LOCI clinics at the study endpoint. This indicates LOCI was more effective than training and technical assistance alone at increasing the sustained uptake of digital measurement-based care 33 months after training, even though these effects were relatively small on a per clinic per month basis. That LOCI was successful in impacting measure administration and clinician viewing of feedback during the sustainment phase is an important signal about the potential of organizational leadership training to improve EBP implementation in mental health settings.

The path to achieving sustained uptake of digital measurement-based care differed by outcome in ways that are consistent with prior studies. Although the control group made some progress increasing the number of youth with a measure administered, these clinics made minimal progress increasing the number of youth with feedback reports viewed by clinicians. This is consistent with findings from measurement-based care effectiveness trials which show that viewing of feedback by clinicians is often infrequent even when measure administration is required and completed regularly [29, 54]. These results suggest training and aligning leaders across organizational levels can do more than merely change intake forms and get measures completed; it can also shift clinical practice in important ways. This is an important finding considering the importance of mental health leaders to implementation efforts [2, 3] and the pronounced lack of evidence-based organizational training available to equip them for success [11, 12].

We offer two hypotheses regarding the precipitous decline in uptake in the LOCI condition prior to its stabilization in the sustainment phase. First, it may be that removal of LOCI terminated the stimulation of the mechanism that explains LOCI’s effect on uptake. For example, leaders may not have been ready to employ leadership skills and strategies without continued LOCI support. This explanation is undermined by research showing that LOCI leaders sustained superior levels of implementation leadership up to six months after the conclusion of LOCI [35]. Another explanation is that a lack of fit between the innovation (digital measurement-based care) and the outer context (e.g. policy, reimbursement) ultimately diminished uptake in (at least some) LOCI clinics [36, 60]. For example, clinic leaders may have used LOCI skills and strategies to rapidly roll-out digital measurement-based care while assessing how well it fit with external reimbursement and policy structures. If leaders perceived a good fit, they may have sustained clinic usage of the system even as other leaders determined it did not fit and discontinued (or never initiated) usage. Because these hypotheses have different implications for improving uptake of digital measurement-based care, trials that test them could generate valuable implications for implementation policy and practice.

Future research should consider ways LOCI might be adapted to increase its effectiveness and scalability in light of this study’s findings. At present, LOCI is designed to address the implementation and sustainment phases of the EPIS model [36], under the assumption that an organization-level adoption decision has already been made. Modifications that expand LOCI’s focus to aid leaders during the exploration and preparation phases may increase the range of implementation outcomes LOCI affects while also strengthening its effects on implementation and sustainment. Another potential modification, currently being studied [61], is to add LOCI components that directly engage system-level leaders, such as policymakers and funders, in an effort to address barriers outside the organization which may influence implementation success.

This research has limitations. Although our outcome measures are robust because they are derived from automated system data, we were unable to examine all aspects of the measurement-based care process, including clinicians’ use of feedback to guide treatment and sharing feedback with youth and caregivers. The large number of datapoints analyzed (*N *= 632 clinic-by-month observations) allowed informative piecewise modeling of changes over time; however, the relatively small number of clinics (*K *= 20) means statistical inferences should be treated with some caution. The correspondence between model-estimated trends and the descriptive patterns observed in the data provides reassurance regarding the substantive results; however, replication in a larger sample would be beneficial. Finally, model inference relies on the assumption that incomplete follow-up data is missing at random conditional on observed covariates included in the analysis; while this assumption cannot be empirically verified, it is plausible given the study design and observed data structure.

Overall, this study confirms that training organizational leaders can meaningfully increase uptake of mental health EBPs across implementation and sustainment phases. It demonstrates the effectiveness of LOCI as a leadership-focused implementation strategy and highlights directions for future research to support leaders in facilitating mental health EBP implementation.

## Supplementary Material

ibag007_Supplementary_Data

## Data Availability

De-identified data from this study are not available in an a public archive. De-identified data from this study will be made available (as allowable according to institutional IRB standards) by emailing the corresponding author.

## Welfare of animals

This article does not contain any studies with animals performed by any of the authors.

## Trial registration

ClinicalTrials.gov Identifier: NCT04096274 (Working to Implement and Sustain Digital Outcome Measures)

## Artificial intelligence

Artificial intelligence was not used in the development of this manuscript.
