# Supplementary material for: Effects of a leadership-focused implementation strategy on uptake of digital measurement-based care in mental health clinics: a cluster randomized trial
Source: Transl Behav Med. 2026 Mar 13;16(1):ibag007. doi: 10.1093/tbm/ibag007 (PMC13016732; doi:10.1093/tbm/ibag007)
Supplement: ibag007_Supplementary_Data [file ibag007_supplementary_data.zip › Supplemental Figure 1.docx.docx]

Supplemental Figure 1. CONSORT diagram showing the flow of clinics through each stage of the trial.

Clinics contacted to assess interest/ eligibility (k=134)

Randomized (k=21)

Allocated to MBC TTA only (k=10)

Allocated to LOCI + MBC TTA (k=11)

Enrollment

Excluded (k=113)

- Did not respond (k=103)
- Did not meet inclusion criteria (k=4)
- Declined to participate (k=6)

Allocation

Follow-Up

Analyzed for primary outcome (k=9)

- Excluded from analysis due to extreme outlier on primary outcome (k=1)

Discontinued study (k=1)

- Clinic closed (k=1)

Discontinued LOCI (k=1)

- Terminated participation (k=1)

Analyzed for primary outcome (k=11)

- Excluded from analysis (k=0)

Analysis

*Note:* LOCI = Leadership and Organizational Change for Implementation strategy (organizational leadership training); MBC = measurement-based care; TTA = training and technical assistance.
