# Supplementary material for: Effects of a leadership-focused implementation strategy on uptake of digital measurement-based care in mental health clinics: a cluster randomized trial
Source: Transl Behav Med. 2026 Mar 13;16(1):ibag007. doi: 10.1093/tbm/ibag007 (PMC13016732; doi:10.1093/tbm/ibag007)
Supplement: ibag007_Supplementary_Data [file ibag007_supplementary_data.zip › Supplemental Table 1.docx.docx]

**Supplemental Table 1.** Leadership and Organizational Change for Implementation (LOCI) Strategy Dosage and Attendance by Clinic and Overall

| Organization Identifier | # Quarterly Organizational Strategy Meetings Completed | # Monthly Progress & Planning Meetings Completed | # Quarterly Leadership Trainings Attended | # Weekly Coaching Calls Attended | # Group Coaching Calls Attended |
| --- | --- | --- | --- | --- | --- |
| 1 | 4 | 2 | 3 | 25 | 2 |
| 2 | 2 | 4 | 4 | 22 | 2 |
| 3 | 3 | 3 | 4 | 21 | 0 |
| 4 | 4 | 4 | 4 | 25 | 0 |
| 5 | 4 | 5 | 4 | 19 | 3 |
| 6 | 4 | 5 | 4 | 21 | 4 |
| 7 | 2 | 2 | 2 | 19 | 2 |
| 8 | 2 | 0 | 4 | 21 | 0 |
| 9 | 3 | 1 | 2 | 25 | 3 |
| 10 | 4 | 4 | 4 | 22 | 0 |
| 11 | 4 | 4 | 4 | 23 | 4 |
| Mean (SD) | 3.3 (0.9) | 3.1 (1.6) | 3.5 (0.8) | 22.1 (2.2) | 1.8 (1.6) |
